# 

**Published:** 1895-02-02

**Authors:** 


PRICE TWOPENCE.

[illegible] 436, Vol. XVII.—Feb. 2nd, 1895.

[illegible]egistered at the General Post Office as a Newspaper.

WITH EXTRA NURSING SUPPLEMENT.

Per Annum, 10s. 6d., post free.

Monthly Parts, 6d.; post free, 8d.

Ditto per Annum, 8s. 6d., post free.

### THE HOSPITAL

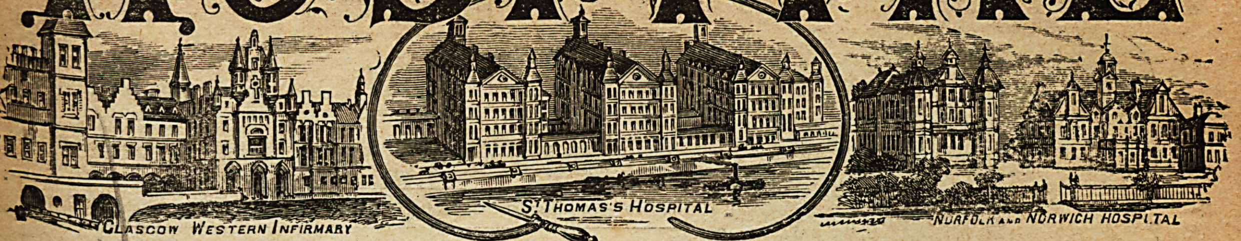

No. 436. Vol. XVII. WITH EXTRA NURSING SUPPLEMENT. SATURDAY, FEB. 2ND, 1895.

